# Association between depressive symptoms and sarcopenia among middle-aged and elderly individuals in China: the mediation effect of activities of daily living (ADL) disability

**DOI:** 10.1186/s12888-024-05885-y

**Published:** 2024-06-10

**Authors:** Qiugui Li, Wenjiao Cen, Tao Yang, Shengru Tao

**Affiliations:** 1 School of Nursing, Jinan University, Guangzhou, Guangdong China; 2 Department of Neurosurgery, the First Affiliated Hospital of Jinan University, Guangzhou, Guangdong China; 3 Department of Healthcare-associated Infection Management, the First Affiliated Hospital of Jinan University, Guangzhou, Guangdong China

**Keywords:** Depressive symptoms, ADL disability, Sarcopenia, logistic regression, Mediation effect

## Abstract

**Background:**

Depressive symptoms and sarcopenia, often observed among middle-aged and elderly individuals, are significant health concerns in China, particularly given the country’s rapidly aging population. Depressive symptoms, characterized by persistent feelings of sadness and loss of interest, can significantly impact quality of life. Little is known about the underlying pathway connecting these two conditions.

**Methods:**

The data for this study were derived from the China Health and Retirement Longitudinal Study (CHARLS). Depressive symptoms were evaluated using the Centre for Epidemiological Studies Depression (CSED) scale. Logistic regression analyses were employed to investigate the association between depressive symptoms, activities of daily living (ADL) disability, and sarcopenia, while adjusting for potential confounding factors. The selection of predictor variables, including social activity, chronic diseases, demographic factors, and lifestyle habits, was based on their known associations with mental health, physical functioning and sarcopenia. These variables were included to ensure a comprehensive adjustment for potential confounding factors and to provide a more accurate estimation of the relationship between depressive symptoms and sarcopenia. Additionally, mediation analysis was conducted to assess the mediating role of ADL disability in the relationship between depressive symptoms and sarcopenia.

**Results:**

A comprehensive study was conducted on a total of 8,238 participants aged 45 years and older, comprising 3,358 men and 4,880 women. Logistic regression analyses were conducted to identify significant associations between depressive symptoms (OR = 1.30, *P* = 0.0269,95%CI = 1.03–1.63), ADL disability (OR = 1.94, *P* < 0.001,95%CI = 1.37–2.75) and sarcopenia. The results revealed significant relationships among these variables. Furthermore, mediation effect analyses demonstrated that ADL disability partially mediated the association between depressive symptoms and sarcopenia (estimated indirect effect: 0.006, 95% CI: 0.003, 0.008, proportion of mediation effect: 20.00%).

**Conclusions:**

The study underscores a significant association between depressive symptoms and sarcopenia among middle-aged and elderly individuals in China, with ADL disability acting as a mediator. These findings offer novel insights for targeted health interventions. Future interventions should effectively combat sarcopenia by integrating psychological support with muscle-strengthening exercise programs. By addressing both depressive symptoms and ADL disability, clinicians and public health professionals can enhance outcomes for this demographic. Collaborative efforts across disciplines are essential for providing comprehensive health management tailored to the needs of middle-aged and elderly individuals. Future research should longitudinally assess the impact of such integrated interventions on sarcopenia prevention and depressive symptom alleviation. Additionally, investigating the role of social and environmental factors in mediating this relationship is crucial for developing more effective health strategies for this vulnerable population.

**Supplementary Information:**

The online version contains supplementary material available at 10.1186/s12888-024-05885-y.

## Introduction

Sarcopenia, a degenerative disease associated with the ageing process, is characterized by a progressive and generalized decline in muscle mass, strength, and somatic function [1]. Multiple studies [2, 3]have demonstrated that muscle mass begins to decline around the age of 30, with an initial loss of 3–8% over the decade. This places middle-aged individuals at an elevated risk of developing sarcopenia. Sarcopenia is linked to numerous adverse outcomes, including disability, falls, fractures, debilitation, compromised health-related quality of life, and increased mortality [4, 5]. With the steady increase in life expectancy, the prevalence of sarcopenia is expected to rise, further exacerbating the disease burden in the decades ahead. This trend holds significant social and economic consequences.

Depression is a mental health disorder characterized by persistent feelings of sadness, hopelessness, loss of interest or pleasure, sleep disturbances and other symptoms [6]. Depression has emerged as a significant mental health challenge among middle-aged and elderly individuals in recent years, with a rise in reported cases in China [7, 8]. These symptoms not only cause psychological distress, physical disability, and family breakdowns but also expedite the progression of chronic diseases and elevate mortality rates in this population [9]. Depressive symptoms are the early signs of depression [10]. For instance, Zakharova and colleagues highlighted a strong association between sarcopenia and depressive symptoms among community-dwelling middle-aged and elderly adults in Japan [11]. Other studies suggest that depressive symptoms may serve as a significant predictor of sarcopenia. Middle-aged and elderly adults experiencing depressive symptoms are at a higher risk of muscle wasting and functional decline, which subsequently increases their vulnerability to falls and other unintentional injuries. Therefore, prompt identification and treatment of depressive symptoms among middle-aged and elderly adults are imperative for preventing sarcopenia and maintaining both physical and mental well-being. Despite the widespread interest in the nexus between depressive symptoms and sarcopenia, there is still limited understanding of the underlying mechanisms driving this relationship.

Activities of Daily Living (ADL) encompass routine tasks that individuals perform independently for self-care, such as eating, dressing, bathing, and mobility [12]. ADL serve as a crucial metric for assessing physical activity and an individual’s capacity to independently care for themselves in performing daily tasks or activities [13–16]. ADL disability refers to impairments or limitations in the ability to independently perform these basic activities of daily life due to health problems or physical functional limitations [17]. Multiple studies have demonstrated a strong association between depressive symptoms and ADL disability [18, 19]. Specifically, ADL disability, the primary cause of functional impairment, refers to an individual’s difficulty in executing essential physical activities required for independent living, often necessitating assistance from others [20]. Depressive symptoms frequently coexist with ADL disability, and the presence of depressive symptoms significantly predicts the subsequent development of ADL disability [21, 22]. A cross-sectional study encompassing 5,863 elderly adults established a link between depressive symptoms and ADL disability among Chinese elderly adults [23]. Additionally, a growing body of research [24–26], indicates that ADL disability may also act as a risk factor for sarcopenia. Factors such as prolonged physical inactivity, malnutrition, and chronic diseases can contribute to the onset of ADL disability, which are also significant risk factors for sarcopenia. Given this overlap, it is plausible that ADL disability mediates the relationship between depressive symptoms and sarcopenia among middle-aged and elderly adults. We hypothesize that ADL disability may act as a potential mediator in the association between depressive symptoms and sarcopenia. This hypothesis is based on the premise that functional limitations in daily activities could worsen depressive symptoms, subsequently contributing to the onset or progression of sarcopenia. Therefore, considering ADL disability as a mediator could offer insights into the complex relationship between depressive symptoms and sarcopenia.

The objectives of this study are twofold: firstly, to investigate the association between depressive symptoms and sarcopenia among middle-aged and elderly Chinese adults; secondly, to explore whether ADL disability serves as a mediator in this relationship. While previous studies have established a longitudinal link between depressive symptoms and sarcopenia, there remains a significant gap in understanding the precise mechanisms underlying this association. Theoretically, the study aims to deepen our understanding of the mechanisms linking depressive symptoms, sarcopenia, and ADL disability, filling a crucial gap in the existing literature. Practically, the findings of this study have the potential to inform the development of targeted interventions for middle-aged and elderly Chinese adults, improving their mental and physical health outcomes. Understanding the relationship between depressive symptoms, ADL disability, and sarcopenia could lead to tailored interventions such as cognitive-behavioral therapy for depressive symptoms, resistance training for sarcopenia, and modifications to living environments to support ADL performance. These interventions could significantly enhance the overall well-being and quality of life for middle-aged and elderly Chinese adults, addressing their specific needs and promoting better mental and physical health outcomes. In addition, recent studies have examined the role of spiritual well-being in predicting hope among elderly populations [27]and the effectiveness of mindfulness-based compassion therapy on sleep quality and life satisfaction among elderly women [28]. These findings provide further insights into the multifaceted nature of mental health and well-being among the elderly.

To address the proposed hypothesis and delve deeper into the intricate relationship among ADL disability, depressive symptoms, and sarcopenia, aiming to understand how these factors interact and influence each other, we utilized data from the China Health and Retirement Longitudinal Study (CHARLS) database. Our study employed a cross-sectional design, and statistical analyses were conducted using mediation analysis techniques to assess the mediation effect of ADL disability.

## Methods

### Data sources

**Fig. 1:**
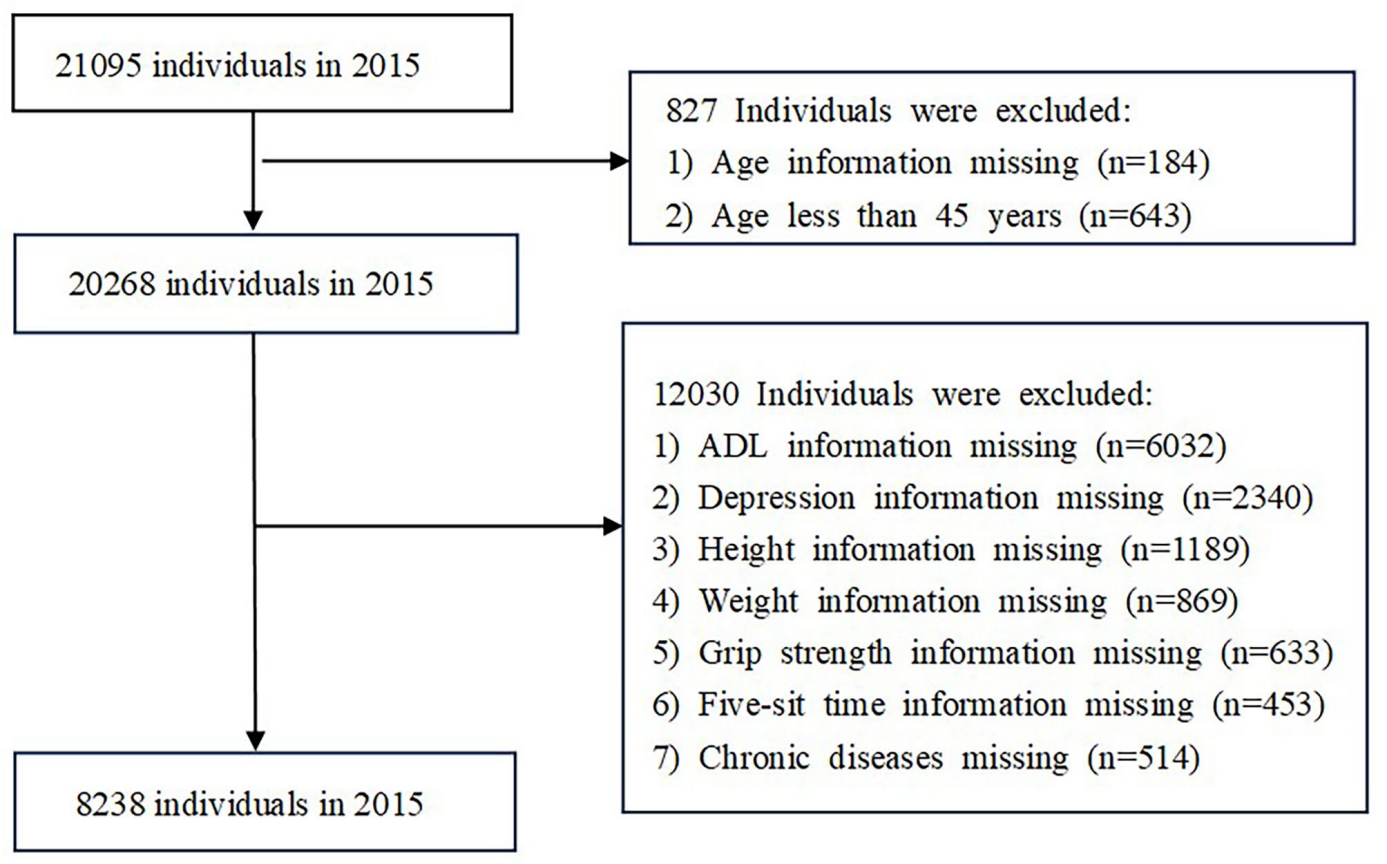
Flow chart of participants through the study

We utilized data from the CHARLS, a publicly accessible dataset accessible via http://charls.pku.edu.cn. CHARLS is a longitudinal survey that tracks individuals aged 45 and above, encompassing 150 counties and 450 municipalities (villages) spread across 28 provinces (autonomous regions and municipalities directly under the central government). The data collection process involved face-to-face interviews conducted by trained interviewers using a standardized protocol. Quality control measures, including rigorous training and supervision, were implemented to ensure consistency across interviews. The sampling approach employed a multi-stage methodology, utilizing probability proportional to size (PPS) sampling at both the district and village levels. Overall, the rigorous sampling frame, inclusion criteria and data collection procedures of CHARLS contribute to the reliability and generalizability of the findings derived from this dataset. The project has received approval from the Biomedical Ethics Committee of Peking University (IRB00001052-11015). All methods employed in this study were conducted in strict accordance with the applicable guidelines and regulations established by CHARLS and in compliance with the principles outlined in the Declaration of Helsinki. Prior to participation, all individuals voluntarily enrolled in CHARLS and provided informed consent by signing a consent form. To safeguard participants’ privacy, confidentiality measures such as data anonymization and restricted access to authorized personnel were implemented. For this study, we conducted an analysis using data from the CHARLS 2015 survey. After excluding participants with missing data, a total of 8238 individuals aged 45 and above were included in our analysis, the specific screening process is shown in Fig. 1. To mitigate potential sources of bias, several strategies were implemented. Firstly, rigorous data cleaning procedures were applied to eliminate inconsistencies and outliers. Secondly, adjusted regression models were utilized to control for confounding variables. This study was carried out in accordance with the AGReMA reporting guideline (Supplement S1 table) [29].

### Data extraction

#### Measurement of sarcopenia

According to the AWGS2019 recommended criteria [30], the assessment of sarcopenia includes muscle strength, physical performance and appendicular skeletal muscle mass. For muscle strength measurements, two tests are performed with the dominant and non-dominant hand, with at least 15 s between each test, and the maximum values of the right and left hands are averaged. The thresholds for low muscle strength were established at less than 28 kg for males and less than 18 kg for females. In terms of physical performance, a decline was indicated if the subject exceeded 12 s to complete 5 sit-ups, exhibited a 6-meter walk speed slower than 1 m per second, or scored below 9 on the Short Physical Performance Battery test. For the measurements of appendicular skeletal muscle (ASM), validated anthropometric equations were employed for estimation [31]. These equations demonstrated excellent concordance with dual energy X-ray absorptiometry (DXA) results. In our study cohort, the cut-off for low muscle mass was determined by the lowest 20% of sex-specific, height-adjusted muscle mass (ASM/Ht^2^). Height and weight were denoted in centimeters and kilograms, respectively, while gender was designated as 1 for males and 2 for females. ASM/Ht^2^ values below 5.08 kg/m^2^ for women and 6.88 kg/m^2^ for men were deemed indicative of low appendicular skeletal muscle mass. The specific ASM equations used are as follows:

$$\begin{gathered}ASM = 0.193*weight\left( {kg} \right) + 0.107*height\left( {cm} \right) \hfill \\\,\,\,\,\,\,\,\,\,\,\,\,\,\,\, - 4.157*gender - 0.037*age\left( {years} \right) - 2.631 \hfill \\ \end{gathered}$$

Sarcopenia is characterized by a combination of low muscle strength, decreased physical performance, or reduced appendicular skeletal muscle mass. A potential diagnosis of sarcopenia can be made based on low muscle strength, with or without concurrent low appendicular skeletal muscle mass. Participants exhibiting low muscle strength, compromised physical performance, and decreased appendicular skeletal muscle mass were considered to have severe sarcopenia. In this study, the participants were categorized into two distinct groups: those with sarcopenia and those without.

#### Measurement of depressive symptoms

To assess depressive symptoms, this study employs the Centre for Epidemiological Studies Depression (CESD) scale. This tool is specifically designed to assess a person’s mood and behavioral patterns related to depressive symptoms over the past week. The frequency of symptoms is categorized into four distinct categories: “Rarely or none of the time”,“Some or a little of the time”,“Occasionally or a moderate amount of the time”,“Most or all of the time”, with corresponding scores of 0, 1, 2, and 3, respectively. Higher total scores on the CESD indicate more severe depressive symptoms. According to the scoring criteria of the scale, a CESD score of 10 or higher indicates the presence of depressive symptoms, suggesting a heightened risk of depression. Conversely, a score below 10 suggests the absence of such symptoms, indicating a lower risk of depression [32].

#### Measurement of ADL disability

The Katz Index of Independence in Activities of Daily Living (Katz ADL) [33] was utilized to assess participants’ ability to carry out routine tasks. The CHARLS questionnaire encompassed six crucial areas: bathing, dressing, toileting, transferring, continence, and feeding; For each of these activities, four options were provided: (1) “No, I don’t have any difficulty”; (2) “I have difficulty but can still do it”; (3) “Yes, I have difficulty and need help” and (4) “I can not do it”. Participants who required help or were unable to accomplish one or more of these six ADL tasks were categorized as having an ADL disability. Conversely, those who demonstrated independence in all six areas were deemed capable of performing their ADL without assistance.

### Measurement of covariates

#### Measurement of social activity

Social activities were gauged using the CHARLS questionnaire, which posed the question, “Have you done any of these activities in the last month? (Code all that apply)”. If a participant engaged in any of the 11 social activities enumerated in the questionnaire, it was recorded as “yes = 1”; otherwise, it was noted as “no = 0”. This binary coding facilitated the counting of the number of social activity items. The questionnaire presented a diverse array of social activities for participants to select from, encompassing various forms of interpersonal engagement. These included: (1) Interacted with friends; (2)Played Ma-jong, played chess, played cards, or went to community club; (3) Provided help to family, friends, or neighbors who do not live with you; (4) Went to a sport, social, or other kind of club; (5) Took part in a community-related organization; (6) Done voluntary or charity work; (7)Cared for a sick or disabled adult who does not live with you; (8) Attended an educational or training course; (9) Stock investment; (10) Used the Internet; (11) Other activities.

#### Measurement of the number of chronic diseases

To ascertain the presence of chronic conditions among participants, we posed the question, “Have you been diagnosed with [conditions listed below, read one by one] by a doctor?” The chronic conditions enumerated in this study encompassed a diverse range of health issues, including hypertension, diabetes or high blood sugar, cancer or malignant tumor, chronic lung diseases such as chronic bronchitis and emphysema, heart attack, stroke, emotional, nervous, or psychiatric problems, arthritis, dyslipidemia, liver disease, kidney disease, stomach or other digestive disorders, and asthma. The participants were then categorized based on the number of chronic diseases they reported, with three distinct groups: those with no chronic conditions, those with one chronic condition and those with two or more chronic conditions.

**Fig. 2:**
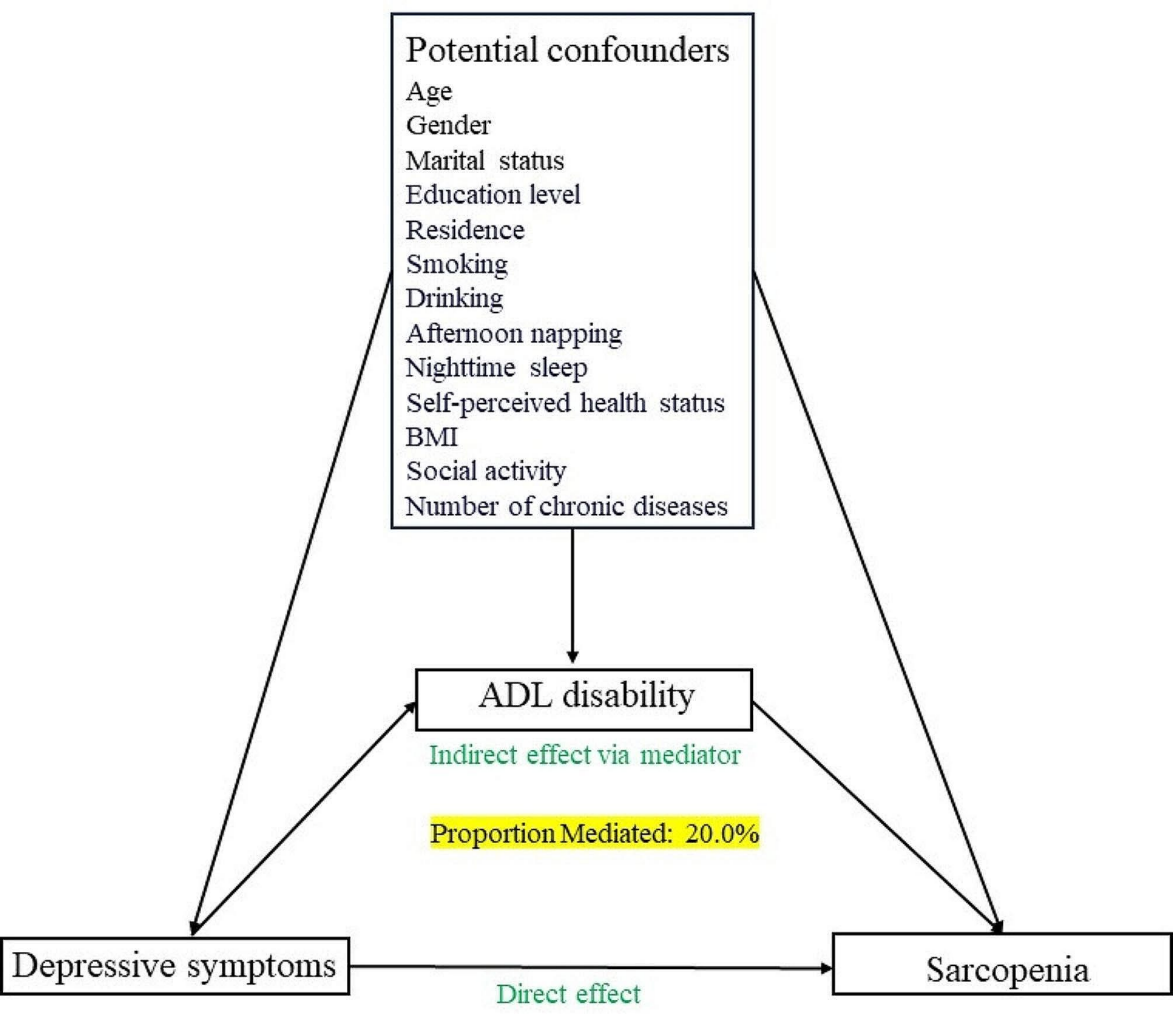
Directed acyclic graph

The other covariates encompassed a broad range of socio-demographic, lifestyle and health-related factors. Socio-demographic factors include gender, age, marital status, education level, residence. Gender is defined as male and female. Education level is categorized as primary school and below, middle school, high school and above. Marital status was defined as married if the person was currently married and living with their spouse; unmarried if the person was currently separated, divorced, widowed or never married. Residence is divided into rural and urban. Lifestyle and health factors included smoking, drinking, afternoon napping, nighttime sleep, self-perceived health status and BMI. Smoking and drinking are defined as “yes” and “no”. Nighttime sleep and afternoon napping were obtained from the questions “During the past month, how many hours of actual sleep did you get at night (average hours for one night?” “During the past month, how long did you take a nap after lunch?”. We categorized nighttime sleep as “<6 h”, “6-9 h” and “≥9h” [34], and afternoon napping as “0min”, “≤60min” and “>60min”. Self-perceived health status categorized as “Good”, “fair” and “poor”. BMI was classified into three categories: underweight (BMI < 18.5 kg/m^2^), normal weight (BMI = 18.5 kg/m^2^ to 24 kg/m^2^) and overweight or obese (BMI ≥ 24 kg/m^2^) [35]. For a comprehensive overview of each variable included in our analysis, please refer to Table 1, which provides detailed information on their definitions, coding, and categorization. The directed circular graph is shown in Fig. 2.

**Table 1:** Coding of variables

Variables	Coding
Gender	Male = 1, Female = 2
Residence	Rural = 1, Urban = 2
Education level	Primary school and below = 1, Middle school = 2, High school and above = 3
Marital status	Married = 1, Unmarried = 2
Self-perceived health status	Good = 1, Fair = 2, Poor = 3
Number of chronic diseases	No chronic disease = 1, One chronic disease = 2, Two or more chronic diseases = 3
Hypertension	No = 0, Yes = 1
Dyslipidemia	No = 0, Yes = 1
Diabetes	No = 0, Yes = 1
Cancer	No = 0, Yes = 1
Chronic lung diseases	No = 0, Yes = 1
Liver disease	No = 0, Yes = 1
Heart diseases	No = 0, Yes = 1
Stroke	No = 0, Yes = 1
Kidney disease	No = 0, Yes = 1
Digestive system diseases	No = 0, Yes = 1
Emotion or mental problems	No = 0, Yes = 1
Memory-related disease	No = 0, Yes = 1
Arthritis	No = 0, Yes = 1
Asthma	No = 0, Yes = 1
Nighttime sleep	< 6 h = 1, 6–9 h = 2, ≥9 h = 3
Afternoon Napping	0 min = 1, ≤ 60 min = 2, > 60 min = 3
Social activity	No = 0, Yes = 1
Smoking	No = 0, Yes = 1
Drinking	No = 0, Yes = 1
Depressive symptoms	No = 0, Yes = 1
BMI	Underweight = 1, Normal weight = 2, Overweight = 3
ADL disability	No = 0, Yes = 1

Abbreviations: ADL: activities of daily living

### Statistical analysis

For this study, 2015 data from the CHARLS database were selected for analysis. For continuous variables, we used median and interquartile range for description and rank sum test for between-group comparisons; for categorical variables, we presented them as percentages and used χ^2^ test or Fisher exact test for between-group comparisons.

Then, to explore the association between depressive symptoms, sarcopenia and ADL disability in middle-aged and elderly Chinese adults, three logistic regression models were used to calculate the odds ratio (OR) and its corresponding 95% confidence interval (95% CI). Model 1 was a crude model. Model 2 was adjusted for gender, age, marital status, residence and education level. Model 3 builds on model 2 by adjusting for chronic diseases, smoking, drinking, nighttime sleep, afternoon napping, self-perceived health status, social activity and BMI.

As part of our in-depth investigation, we conducted a mediation analysis using the ‘Bruce’ package in R (https://cran.r-project.org/web/packages/bruceR/bruceR.pdf). This analysis employed a quasi-Bayesian Monte Carlo simulation method, with 1000 iterations, to accurately assess the mediating role of ADL disability between depressive symptoms and sarcopenia in middle-aged and elderly adults. Mediation analysis is a statistical technique that explores how a variable (mediator) intervenes in the relationship between two other variables. This approach aids in understanding the indirect effect of one variable on another through the mediator, offering insights into the underlying mechanisms of complex relationships. It’s important to note that mediation analyses in cross-sectional studies, as discussed by Hayes, are exploratory in nature. Therefore, the mediating effects reported in this study should be interpreted with caution and considered hypothesis-generating for future longitudinal or experimental studies. Throughout the study, we utilized R 4.3.2 software (https://www.r-project.org/) to conduct all statistical analyses. All tests were two-tailed, with statistical significance set at *P* < 0.05.

## Results

### General characteristics of the participants

Of the 8238 participants, the median age was 61 years (interquartile range: 53–68 years), 4880 (59.2%) were female, 3358 (40.8%) were male, 6404 (77.7%) lived in rural areas, 5640 (68.5%) had a primary school education or below, 7049 (85.6%) were married, 4587 (55.7%) had two or more chronic diseases and 4587 (55.7%) had one chronic disease,3471 (42.1%) were experiencing depressive symptoms, 580 (7.0%) had ADL disability, and the prevalence of sarcopenia was 7.68%. The occurrence of sarcopenia is associated with age, residence, education level, marital status, self-perceived health status, chronic disease、nighttime sleep、afternoon napping、social activity、depressive symptoms、BMI、ADL disability. Older age, rural residence, lower educational level, unmarried status, poor self-perceived health status, lower participation in social activities, depressive symptoms, hypertension, dyslipidemia, diabetes, chronic lung disease, ADL disability, abnormal nighttime sleep duration (too long or too short), and poor napping habits (lack of napping or excessive napping time longer than 60 min) significantly increased participants’ risk of sarcopenia (Table 2).

**Table 2:** Baseline of characteristics of participants by sarcopenia groups

Variables	Overall(*n* = 8238)	Non-sarcopenia(*n* = 7605)	Sarcopenia(*n* = 633)	*p*
Age (years), mean (SD)	61.00(53.00,68.00)	61.00(53.00,67.00)	72.00(67.00,78.00)	< 0.001
Gender (%)				0.120
Male	3358 (40.8)	3081 (40.5)	277 (43.8)	
Female	4880 (59.2)	4524 (59.5)	356 (56.2)	
Residence (%)				< 0.001
Rural	6404 (77.7)	5843 (76.8)	561 (88.6)	
Urban	1834 (22.3)	1762 (23.2)	72 (11.4)	
Education level (%)				< 0.001
Primary school and below	5640 (68.5)	5088 (66.9)	552 (87.2)	
Middle school	1956 (23.7)	1893 (24.9)	63 (10.0)	
High school and above	642 (7.8)	624 (8.2)	18 (2.8)	
Marital status (%)				< 0.001
Married	7049 (85.6)	6622 (87.1)	427 (67.5)	
Unmarried	1189 (14.4)	983 (12.9)	206 (32.5)	
Self-perceived health status (%)				< 0.001
Good	1246 (15.1)	1163 (15.3)	83 (13.1)	
Fair	4178 (50.7)	3923 (51.6)	255 (40.3)	
Poor	2814 (34.2)	2519 (33.1)	295 (46.6)	
Number of chronic diseases (%)				0.612
No chronic disease	1568 (19.0)	1456 (19.1)	112 (17.7)	
One chronic disease	2083 (25.3)	1916 (25.2)	167 (26.4)	
Two or more chronic diseases	4587 (55.7)	4233 (55.7)	354 (55.9)	
Hypertension (%)				< 0.001
No	5178 (62.9)	4725 (62.1)	453 (71.6)	
Yes	3060 (37.1)	2880 (37.9)	180 (28.4)	
Dyslipidemia (%)				< 0.001
No	6830 (82.9)	6253 (82.2)	577 (91.2)	
Yes	1408 (17.1)	1352 (17.8)	56 (8.8)	
Diabetes (%)				0.003
No	7369 (89.5)	6780 (89.2)	589 (93.0)	
Yes	869 (10.5)	825 (10.8)	44 (7.0)	
Cancer (%)				0.803
No	8111 (98.5)	7489 (98.5)	622 (98.3)	
Yes	127 (1.5)	116 (1.5)	11 (1.7)	
Chronic lung diseases (%)				< 0.001
No	6884 (83.6)	6432 (84.6)	452 (71.4)	
Yes	1354 (16.4)	1173 (15.4)	181 (28.6)	
Liver disease (%)				0.632
No	7794 (94.6)	7192 (94.6)	602 (95.1)	
Yes	444 (5.4)	413 (5.4)	31 (4.9)	
Heart diseases (%)				0.556
No	6609 (80.2)	6095 (80.1)	514 (81.2)	
Yes	1629 (19.8)	1510 (19.9)	119 (18.8)	
Stroke (%)				0.525
No	7989 (97.0)	7372 (96.9)	617 (97.5)	
Yes	249 (3.0)	233 (3.1)	16 (2.5)	
Kidney disease (%)				0.357
No	7498 (91.0)	6915 (90.9)	583 (92.1)	
Yes	740 (9.0)	690 (9.1)	50 (7.9)	
Digestive system diseases (%)				0.059
No	5871 (71.3)	5441 (71.5)	430 (67.9)	
Yes	2367 (28.7)	2164 (28.5)	203 (32.1)	
Emotion or mental problems (%)				0.822
No	7985 (96.9)	7370 (96.9)	615 (97.2)	
Yes	253 (3.1)	235 (3.1)	18 (2.8)	
Memory-related disease (%)				0.012
No	8030 (97.5)	7423 (97.6)	607 (95.9)	
Yes	208 (2.5)	182 (2.4)	26 (4.1)	
Arthritis (%)				0.622
No	4719 (57.3)	4350 (57.2)	369 (58.3)	
Yes	3519 (42.7)	3255 (42.8)	264 (41.7)	
Asthma (%)				< 0.001
No	7685 (93.3)	7136 (93.8)	549 (86.7)	
Yes	553 (6.7)	469 (6.2)	84 (13.3)	
Nighttime sleep (%)				< 0.001
1	2844 (34.5)	2592 (34.1)	252 (39.8)	
2	4635 (56.3)	4328 (56.9)	307 (48.5)	
3	759 (9.2)	685 (9.0)	74 (11.7)	
Afternoon Napping (%)				0.002
1	3496 (42.4)	3189 (41.9)	307 (48.5)	
2	3226 (39.2)	3018 (39.7)	208 (32.9)	
3	1516 (18.4)	1398 (18.4)	118 (18.6)	
Social activity (%)				< 0.001
No	3781 (45.9)	3426 (45.0)	355 (56.1)	
Yes	4457 (54.1)	4179 (55.0)	278 (43.9)	
Smoking (%)				0.064
No	6193 (75.2)	5737 (75.4)	456 (72.0)	
Yes	2045 (24.8)	1868 (24.6)	177 (28.0)	
Drinking (%)				0.062
No	5723 (69.5)	5262 (69.2)	461 (72.8)	
Yes	2515 (30.5)	2343 (30.8)	172 (27.2)	
Depressive symptoms (%)				< 0.001
No	4767 (57.9)	4459 (58.6)	308 (48.7)	
Yes	3471 (42.1)	3146 (41.4)	325 (51.3)	
BMI (%)				< 0.001
Underweight	509 (6.2)	240 (3.2)	269 (42.5)	
Normal weight	3800 (46.1)	3439 (45.2)	361 (57.0)	
Overweight	3929 (47.7)	3926 (51.6)	3 (0.5)	
ADL disability (%)				< 0.001
No	7658 (93.0)	7121 (93.6)	537 (84.8)	
Yes	580 (7.0)	484 (6.4)	96 (15.2)	
Handgrip strength, kg	26.50 (20.50, 33.25)	27.15 (21.50, 34.00)	17.75 (12.75, 23.75)	< 0.001
ASM/Ht^2^, kg/m2	6.69 (5.87, 7.51)	6.81 (5.96, 7.58)	5.03 (4.68, 6.54)	< 0.001
Gait speed, m/s	1.28 (1.08, 1.56)	1.26 (1.07, 1.53)	1.53 (1.25, 1.97)	< 0.001
5-time chair stand test, s	9.33 (7.63, 11.60)	9.17 (7.53, 11.28)	12.35 (9.57, 15.00)	< 0.001

Abbreviations: ADL: activities of daily living; ASM: appendicular skeletal muscle

Note: Medians and interquartile ranges (25th and 75th percentiles) were calculated for continuous variables and frequencies and percentages for categorical variables. The Wilcoxon rank sum test was used to compare group differences for continuous variables and Chi-squared tests for categorical variables

### Association of depressive symptoms, ADL disability and sarcopenia

To assess the association between depressive symptoms, ADL disability and sarcopenia, we employed logistic regression analysis to determine the odds ratio (OR) along with its corresponding 95% confidence interval (CI). As demonstrated in Table 3, our findings indicate that depressive symptoms (OR = 1.30, 95% CI = 1.03, 1.63, *P* = 0.027) and ADL disability (OR = 1.94, 95% CI = 1.37, 2.75, *P* < 0.001) serve as significant risk factors for sarcopenia among middle-aged and elderly adults.

**Table 3:** Logistic regression analysis of depressive symptoms, ADL disability and sarcopenia

Variables	Model 1^a^		Model 2^b^		Model 3^c^	
	OR (95%CI)	*P*	OR (95%CI)	*P*	OR (95%CI)	*P*
Depressive symptoms	1.39(1.18,1.64)	< 0.001	1.57(1.31,1.88)	< 0.001	1.30(1.03,1.63)	0.027
ADL disability	2.44(1.92,3.09)	< 0.001	1.57(1.20,2.04)	0.001	1.94(1.37,2.75)	< 0.001

Abbreviations: ADL: activities of daily living; OR: odds ratios; CI: confidence intervals

a. A crude model

b. Adjusted for gender, age, marital status, residence, education level)

c. Adjusting for chronic diseases, smoking, drinking, nighttime sleep, afternoon napping, self-perceived health status, social activity, BMI

### Mediating effect of ADL disability between depressive symptoms and sarcopenia

As presented in Table 4, our findings revealed a statistically significant mediating role of ADL disability, with a 95% CI that did not overlap with zero. Additionally, we observed a significant direct effect, indicating that ADL disability partially mediates the association between depressive symptoms and sarcopenia. The estimated indirect effect was 0.006, with a 95% CI ranging from 0.003 to 0.008. The proportion of the mediating effect attributed to ADL disability was 20.00%, further confirming its significant role in the relationship between depressive symptoms and sarcopenia(*P* < 0.001).

**Table 4:** Mediation effects of ADL disability between depressive symptoms and sarcopenia

Effect Type	Path	Effect (95% CI)	Mediating Effect (%)	*P*
Total Effect	Depressive symptoms→Sarcopenia	0.030(0.017,0.042)		< 0.001
Direct Effect	Depressive symptoms→Sarcopenia	0.024(0.012,0.037)	80.00	< 0.001
Indirect Effect	Depressive symptoms→ADL disability→Sarcopenia	0.006(0.003,0.008)	20.00	< 0.001

Abbreviations: ADL: activities of daily living

## Discussion

The current cross-sectional study reveals significant associations between depressive symptoms, ADL disability, and sarcopenia among middle-aged and elderly Chinese adults. Notably, we found that ADL disability partially mediates the relationship between depressive symptoms and sarcopenia. Our study builds upon prior research confirming the link between depressive symptoms and sarcopenia, as evidenced by Han [36] and Zhang [37]. However, our unique contribution lies in investigating the mediating role of ADL disability, offering a deeper understanding of the pathways connecting depression and sarcopenia. While earlier studies primarily focused on the direct association between depressive symptoms and sarcopenia, our research delves into the mediation effect of ADL disability. Overall, our findings provide valuable insights into the mediating role of ADL disability in the depression-sarcopenia pathway, thereby contributing to the existing literature. These insights are pertinent for healthcare providers, policymakers, and public health practitioners, as they inform potential interventions such as enhanced screening, early intervention programs, promotion of physical activity and healthy lifestyles, and tailored support and education for middle-aged and elderly populations. These strategies hold promise for improving health outcomes and enhancing the quality of life for this vulnerable demographic.

Sarcopenia, commonly associated with aging, affects a significant portion of the middle-aged and elderly population worldwide, with prevalence ranging from 6 to 12%. Our study aligns with global statistics, showing a prevalence of 7.68% in this demographic, highlighting the widespread occurrence and importance of this condition [38].It is crucial to raise awareness and provide targeted interventions for vulnerable age groups to address this geriatric syndrome. Our study reveals a higher prevalence of sarcopenia among older individuals, those in rural areas, with lower education levels, unmarried status, poor self-perceived health, limited social activities, depression, hypertension, dyslipidemia, diabetes, chronic lung disease, ADL disability, abnormal sleep duration, and poor napping habits. These factors impact muscle health through various biological, psychosocial, and environmental pathways. Limited access to healthcare and health information in rural areas exacerbates the muscle decline associated with aging [39]. Insufficient awareness of muscle health among the general population is also influenced by limited healthcare resources and lack of health information in rural areas, as supported by recent studies [40, 41]. Marital status [42], self-perceived health [43] and social activities [44] affect lifestyle and psychological well-being, which in turn impact muscle health. Depressive symptoms can further worsen muscle health by reducing appetite, decreasing exercise motivation, disrupting the neuroendocrine system, impairing muscle synthesis, and accelerating catabolism [45–48]. Chronic conditions like hypertension and diabetes alter metabolism, affecting muscles. Abnormal sleep patterns disrupt recovery processes, while poor napping habits lower energy levels and hinder muscle recovery [49, 50]. And sleep quality may also affect pain, a relevant variable in the association between depressive symptoms and sarcopenia [51].

Our study finds that ADL disability increases the risk of sarcopenia by 1.9 times, highlighting the need for targeted interventions. ADL disability limits muscle stimulation and exercise opportunities, accelerating muscle degeneration. Additionally, it is often linked to other health issues like chronic illness and pain, further exacerbating sarcopenia risk. Furthermore, ADL disability can impact psychological factors like self-esteem and social skills, indirectly affecting muscle health. Overall, addressing ADL capacity holistically is crucial for preventing and treating sarcopenia, requiring targeted interventions to improve muscle health and quality of life [52–54].

Our study underscores the pivotal role of ADL disability in connecting depressive symptoms to sarcopenia, suggesting that depressive symptoms may precipitate ADL disability, consequently elevating the risk of sarcopenia. Notably, ADL disability contributes significantly to this association, explaining 20% of the relationship, thus emphasizing its clinical significance. Prior research [55, 56] consistently establish a connection between depressive symptoms and ADL disability, revealing a higher prevalence of ADL disability among individuals experiencing such symptoms. This bidirectional relationship underscores the imperative for comprehensive strategies addressing depression, ADL disability, and sarcopenia in middle-aged and elderly populations, ultimately enhancing overall well-being and quality of life. The observed associations hold clinical significance, notably the substantial 20% contribution of ADL disability to the relationship between depressive symptoms and sarcopenia. This suggests that interventions targeting ADL disability could potentially mitigate a significant portion of sarcopenia risk among depressed individuals. Consequently, early identification and intervention for depressive symptoms, along with the promotion of self-care abilities and ADL functioning, are crucial in this population. In comparison to previous studies [57, 58], our findings align with established links between depressive symptoms and ADL disability while shedding further light on the role of ADL disability in sarcopenia development. It is crucial to consider the magnitude of these associations, as they hold clinical significance, underscoring the need for targeted interventions. Furthermore, comparing these findings with previous studies aids in understanding the consistency and robustness of these associations across diverse populations and settings. Moreover, the Biopsychosocial Model offers a holistic framework for comprehending the interplay between biological, psychological, and social factors in health and illness. According to this model, depression may influence ADL disability through various pathways. For example, it can diminish motivation, energy levels, and interest in self-care activities, thereby impairing an individual’s ability to perform ADL tasks. Moreover, depression may disrupt sleep patterns and appetite, further worsening physical disabilities associated with ADL tasks. Incorporating this model into our understanding allows for a more comprehensive approach to addressing the complexities of depression, ADL disability, and sarcopenia.

Our study has several strengths. Firstly, it is the first to utilize national CHARLS survey data from a representative sample of middle-aged and elderly adults in China to investigate the association between depressive symptoms, ADL disability, and sarcopenia. This dataset offers unique coverage and national representativeness, enhancing the reliability of our findings for the entire Chinese middle-aged and elderly population. Secondly, we adhered to the AWGS guidelines for sarcopenia measurement, which are more tailored to Asian muscle characteristics. This ensures the relevance and accuracy of our results in reflecting muscle health among Chinese middle-aged and elderly individuals. Additionally, our study goes beyond examining the direct relationships between depressive symptoms, ADL disability, and sarcopenia by delving into the mediating role of ADL disability. This comprehensive approach provides deeper insights into the underlying mechanisms of this complex relationship, laying a stronger foundation for targeted interventions.

This study has several limitations. Firstly, we utilized anthropometric equations instead of more advanced methods like DXA or bioelectrical impedance analysis (BIA) for measuring sarcopenia, potentially impacting result accuracy and generalizability. Future research should prioritize DXA or BIA to obtain more precise sarcopenia measurements, enhancing the foundation for investigating the relationships between depressive symptoms, ADL disability, and sarcopenia. Secondly, self-reporting in our survey data introduces subjectivity and bias, although previous studies have validated the measurement accuracy of these variables [59]. Future research should focus on minimizing biases through methodological refinements or incorporating objective measures. Thirdly, we did not include diet and nutritional status as covariates [60–62], limiting our understanding of their potential impact on depressive symptoms, ADL disability, and sarcopenia. Including these factors in future research would enable a more nuanced analysis. Fourthly, our findings may not directly apply to other ethnic or geographic groups due to the specific characteristics of Chinese middle-aged and older adults. Caution is needed when generalizing our results. Future research should aim to validate these relationships in diverse populations to ensure broader applicability and relevance. Finally, because our study was cross-sectional, we were unable to fully investigate the causal relationships underlying the associations. Future research could include longitudinal studies or intervention trials to delve into causal relationships and assess intervention effectiveness in mitigating sarcopenia risk among middle-aged and elderly populations. Longitudinal studies would provide insights into sarcopenia development over time, while intervention trials could evaluate specific interventions’ impact on sarcopenia incidence and progression. These endeavors are essential for advancing understanding and guiding strategies for healthy aging and improved quality of life.

## Conclusions

In response to this finding, future research could further explore how depressive symptoms increases the risk of sarcopenia by affecting ADL disability. For example, research could focus on how ADL change over time in people with depressive symptoms and how this change is related to the development of sarcopenia. In addition, research can explore how effective interventions can improve ADL disability in patients with depressive symptoms, thereby reducing their risk of developing sarcopenia. In summary, the association between depressive symptoms, ADL disability and sarcopenia provides us with new perspectives for understanding and treating these health problems. By understanding these relationships, we can develop more effective methods and strategies to prevent and treat these diseases.

### Electronic supplementary material

Below is the link to the electronic supplementary material.

AGReMA Checklist

## Data Availability

The datasets generated during and/or analysed during the current study are available in the CHARLS repository, http://charls.pku.edu.cn.
