# Supplementary figures and images for: Association between depressive symptoms and sarcopenia among middle-aged and elderly individuals in China: the mediation effect of activities of daily living (ADL) disability
Source: BMC Psychiatry. 2024 Jun 10;24:432. doi: 10.1186/s12888-024-05885-y (PMC11165901; doi:10.1186/s12888-024-05885-y)

S1 table AGReMA Checklist


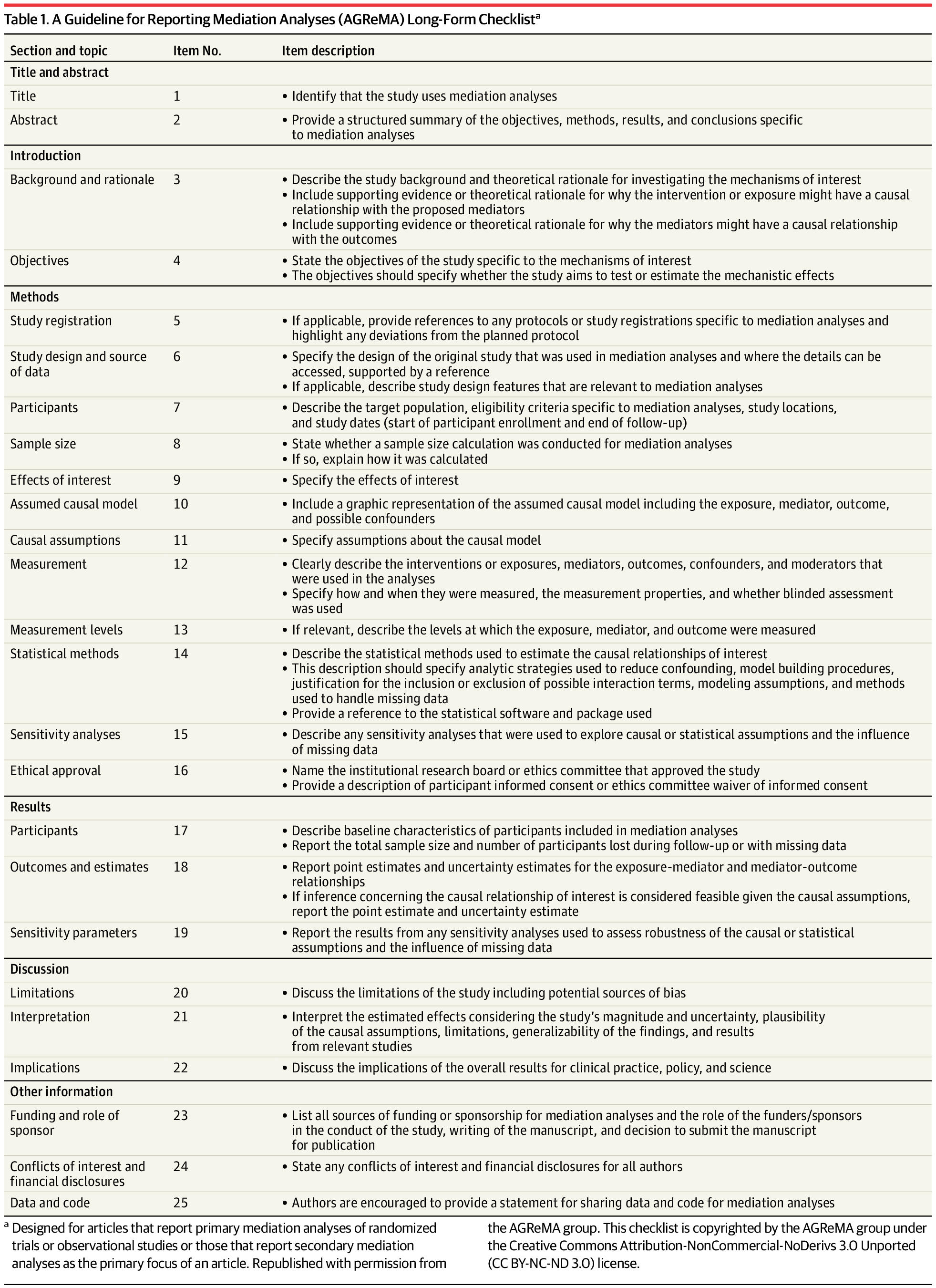

Supplement: Supplementary file 1 — AGReMA Checklist [file 12888_2024_5885_MOESM1_ESM.docx]
